# Supplementary material for: The Possible Mechanism of Amyloid Transformation Based on the Geometrical Parameters of Early-Stage Intermediate in Silico Model for Protein Folding
Source: Int J Mol Sci. 2022 Aug 22;23(16):9502. doi: 10.3390/ijms23169502 (PMC9409474; doi:10.3390/ijms23169502)
Supplement: Supplementary file 1 [file ijms-23-09502-s001.zip › ijms-1843474-supplementary.pdf]

SUPPLEMENTARY MATERIALS

The possible mechanism of amyloid transformation based on the geometrical parameters of early-stage intermediate in silico model for protein folding

Roterman I, Stapor K, Dułak D, Konieczny L.

Early intermediate model in the protein folding process based on the geometrical parameters: radius of curvature R and angle V expressing the rotation of the planes of adjacent peptide bonds

The specificity of the elliptical path for the generation of early intermediate structures in the model reflecting the protein folding process is visualized in Figure. The structures highlighted in Figure S1 reveal the transition from the R-helix (parallel hydrogen bond orientation - Figure S1.A) through the structure with the Psi = - Phi characteristic (Figure S1.G) with the anti-parallel hydrogen bond system in Beta-structural form and reoccurrence of a parallel system from the L-helix case (Figure S1.M). Structures visualize the distinguished dark dots on the elliptical path on the Ramachandan map (Figure S1.O). The main point of this presentation is the tracing of mutual orientations of C=O groups as potential participants in hydrogen bonds system generation (red arrows on the Figure S1).

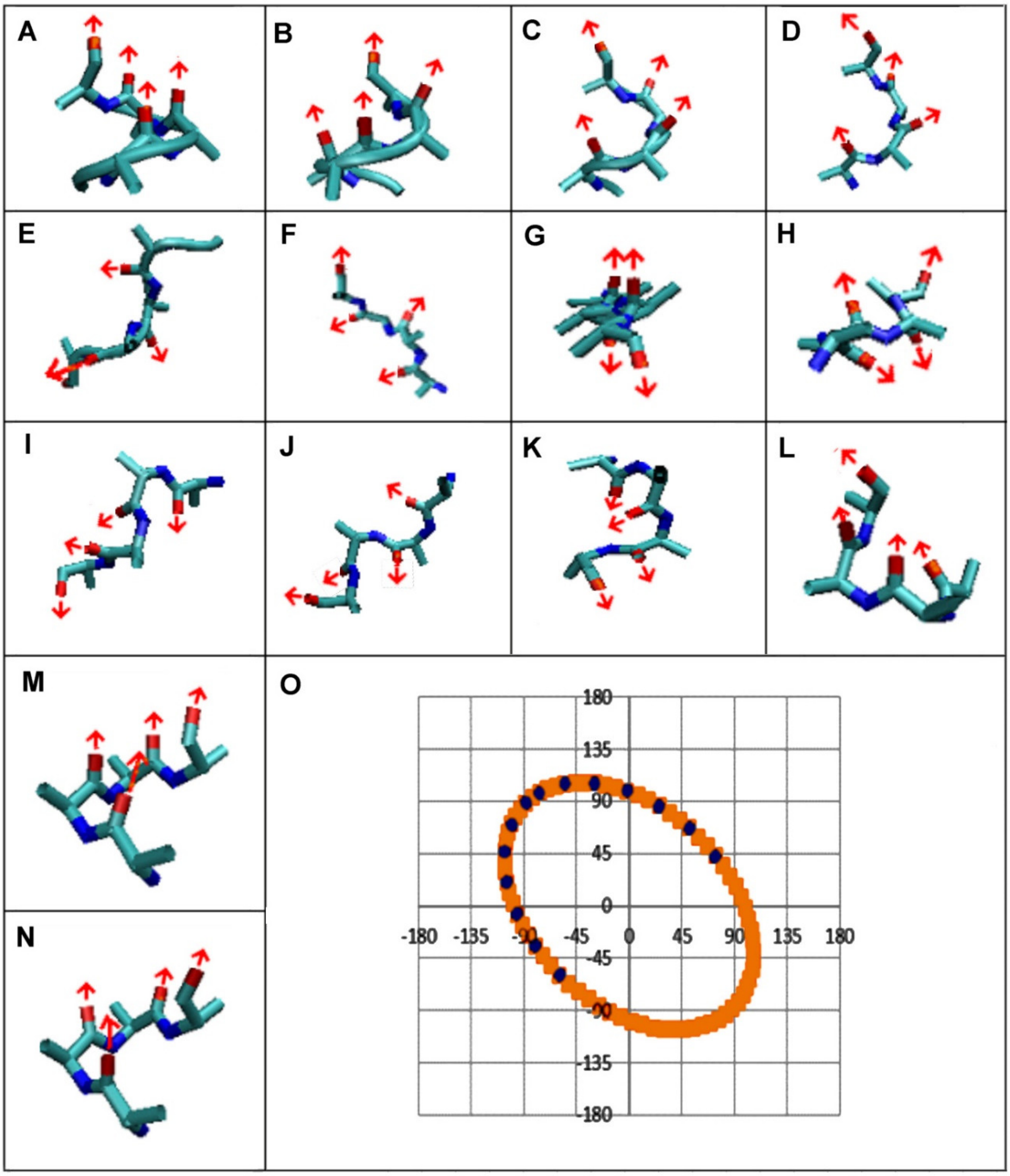

Figure S1. The list of 3D structures on the path (A-N – starting at 7 o'clock and going clock-wise according to Figure S1.O). The red arrows reveal the variability of the orientation of the planes of peptide bonds visualized by the orientation of C=O bonds

Introduction to structural codes

The elliptical path representing a limited conformational subspace for an early intermediate in the protein folding process gives rise to the structural codes. These codes were obtained by projecting the Phi and Psi angles (as observed in native proteins in the nonredundant PDB base) onto elliptical path resulting in Phie and Psie angles (index e means belonging to an elliptical path). Projecting is done on the principle of the shortest

distance between Phi and Psi and the elliptical path. The frequency profile of the corresponding Phie and Psie conformations reveals the presence of the seven local peaks (Figure S2). By assigning an appropriate code to each of the observed local maximums, it is possible to distinguish zones representing the local maxima on the Ramachandran map (Figure S3).

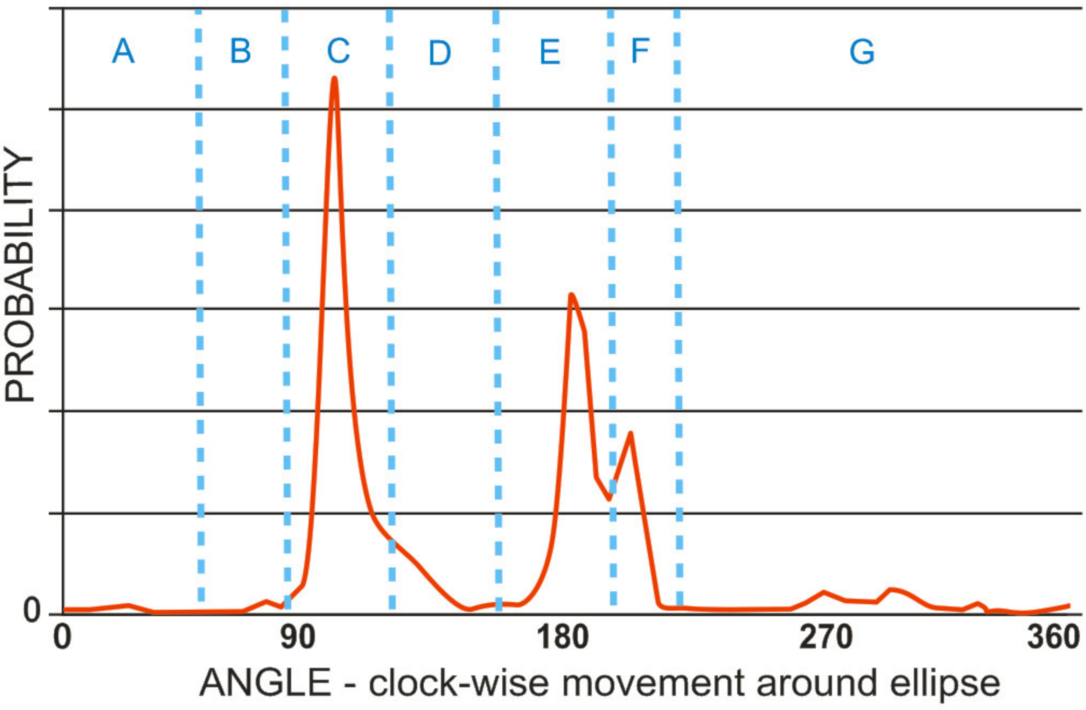

Figure S2. Profile representing the probability distribution of the Phie and Psie angles, determined on the basis of the non-redundant PDB analysis [Fabian P, Stapor K, Roterman I. Model of Early Stage Intermediate in Respect to Its Final Structure. Biomolecules 2019 ; 9(12):866. doi: 10.3390/biom9120866.]

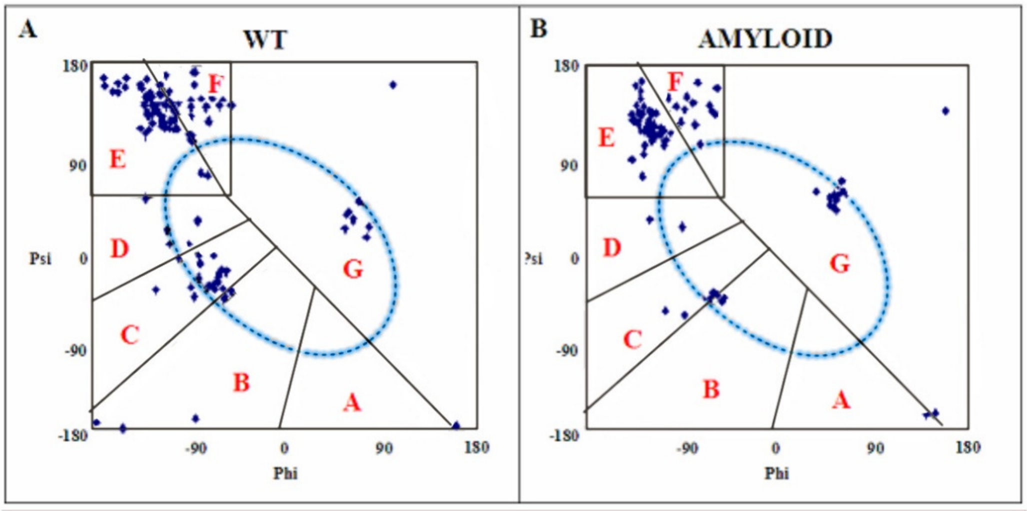

Figure S3. Distribution of the Phi and Psi angles

A - WT form of transthyretin (1DVQ)

B – amyloid form of transthyretin

in relation to the designated zones consistent with the probability distribution (Figure S2).

Importance of E and F zones distinction in amyloid analysis

In the analysis of the amyloid structure, the distinction between the E and F zones is important. Beta-structure is treated in the secondary structure classification as one common class containing both E and F zones. However this distinction between E and F zone appears important in the light of the presented analysis as the specific factor in amyloid analysis. It results from different geometric parameters. For the E form, the structures are characterized by high radius of curvature R and V-angle values close to 180 deg. In contrast, zone F shows lower radius of curvature and lower values of V-angle. The common treatment of these two structural forms results from the fact that the terminal sections of the Beta structure are characterized by a reduction in the radius of curvature. Otherwise, the rectilinear form would propagate no limit. Each beta-structure segment terminates a loop segment with a significantly reduced radius of curvature. Since these elements of the termination of the Beta structure accompany the Beta structure, it is reasonable to consider them together. However, from the form of the two-peak local maximum in the distribution of structures along the elliptical path, it should be concluded that the forms E and F are different and need to be differentiated (Figure S2 and Figure S3).

As shown in the analysis of transthyretin in its amyloid form (as well in other amyloids available in PDB) in the main body of this work, this distinction is justified by the reduction in the presence of the F form in the amyloid form. This is due to the fact that the termination of beta-structural segments is different than in native proteins. In amyloid it does not take the form of a gently tapering radius of curvature causing loops to appear as is the case with native forms of proteins. In the case of amyloids, termination of Beta-structural segments is radical in the form of the introduction of a single residue with an R- or L-helix conformation.

For the visualization of the variability of the geometric form of the polypeptide during the transition from form F to E, the conformational changes for the transition from Phi = 0 and Psi = 150 deg to Phi = - 150 and Psi = 150 were visualized. The structures in figures Figure S4A to Figure S4E visualize the G zone, where the radius of curvature is rather small becoming bigger and bigger step-wise reaching higher values of radius of curvature in F zone (Figure S4F to Figure S4I). However the linear form with antiparallel orientation of hydrogen bonds is present in structures belonging to E zone. It is visualized in Figure S4O-S4S where the antiparallel orientation of hydrogen bonds is observed. This form of organization is observed in amyloid under consideration.

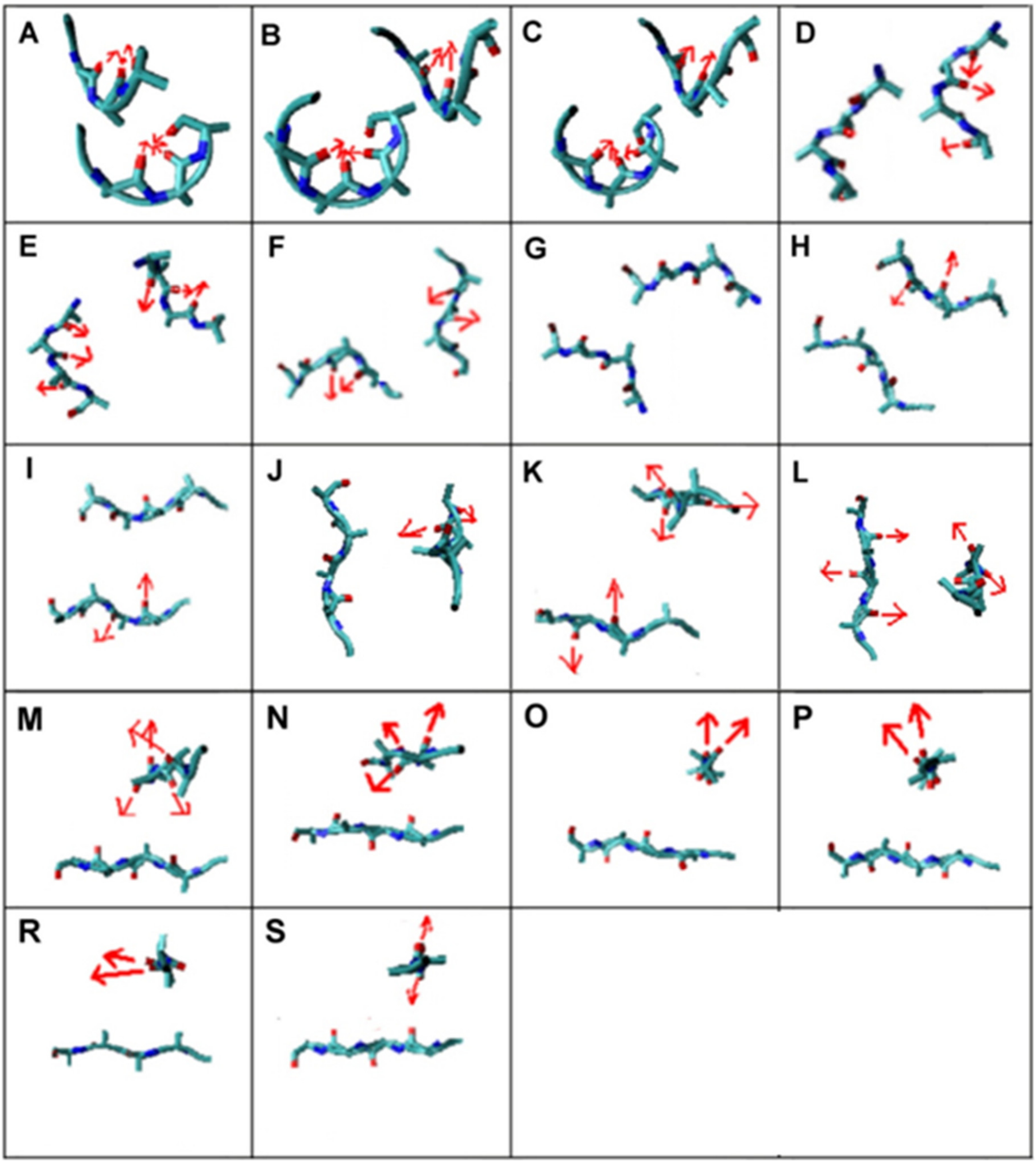

Figure S4. The structural forms generated according to the distinguished points on Ramachandran map (Figure S5) to visualise the changes of geometrical parameters : radius of curvature and V-angle following the path distinguished on Figure 5S – dark blue points. It is aimed to show significant differences between E and F zone on Ramachandran map (Figure S3).

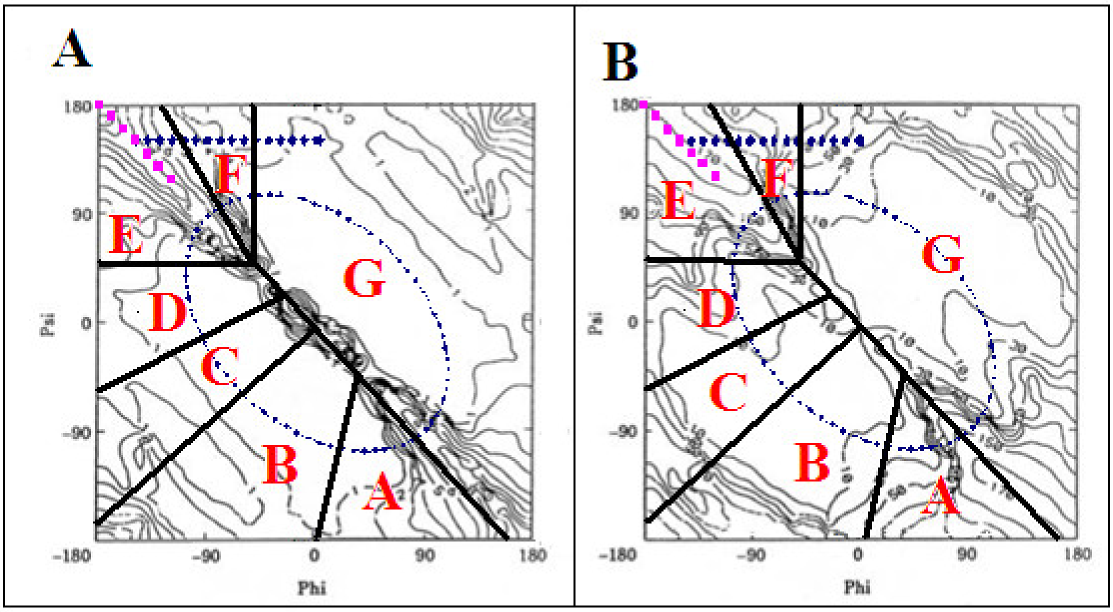

Figure S5. The selected conformations visualised in Figure S4. The set of dark-blue points is distinguished to show the structure changes following the F to E zone transition.

A – the selected path in respect to radius of curvature (in ln(R) scale) distrubution on the Ramachandran map

B - the selected path in respect to V-angle distrubution on the Ramachandran map

Pink dots – the conformations present in amyloid form with highest radius of curvature and V-angle = 180 degs allowing the construction of hydrogen bond system in antiparallel orientation.
